# Computer-aided detection in chest radiography based on artificial intelligence: a survey

**DOI:** 10.1186/s12938-018-0544-y

**Published:** 2018-08-22

**Authors:** Chunli Qin, Demin Yao, Yonghong Shi, Zhijian Song

**Affiliations:** 1 School of Basic Medical Sciences, Digital Medical Research Center, Fudan University, Shanghai, China; 2 Shanghai Key Laboratory of Medical Imaging Computing and Computer Assisted Intervention, Shanghai, China

**Keywords:** Artificial intelligence, Computer-aided detection, Chest radiography, Disease classification

## Abstract

As the most common examination tool in medical practice, chest radiography has important clinical value in the diagnosis of disease. Thus, the automatic detection of chest disease based on chest radiography has become one of the hot topics in medical imaging research. Based on the clinical applications, the study conducts a comprehensive survey on computer-aided detection (CAD) systems, and especially focuses on the artificial intelligence technology applied in chest radiography. The paper presents several common chest X-ray datasets and briefly introduces general image preprocessing procedures, such as contrast enhancement and segmentation, and bone suppression techniques that are applied to chest radiography. Then, the CAD system in the detection of specific disease (pulmonary nodules, tuberculosis, and interstitial lung diseases) and multiple diseases is described, focusing on the basic principles of the algorithm, the data used in the study, the evaluation measures, and the results. Finally, the paper summarizes the CAD system in chest radiography based on artificial intelligence and discusses the existing problems and trends.

## Background

Chest radiography (chest X-ray or CXR) is an economical and easy-to-use medical imaging and diagnostic technique. The technique is the most commonly used diagnostic tool in medical practice and has an important role in the diagnosis of the lung disease [1]. Well-trained radiologists use chest X-rays to detect illnesses, such as pneumonia, tuberculosis, interstitial lung disease, and early lung cancer.

The great advantages of chest X-rays include their low cost and easy operation. Even in underdeveloped areas, modern digital radiography (DR) machines are very affordable. Therefore, chest radiographs are widely used in the detection and diagnosis of the lung diseases, such as pulmonary nodules, tuberculosis, and interstitial lung disease. Chest radiography contains a large amount of information about a patient’s health. However, correctly interpreting the information is always a major challenge for the doctor. The overlapping of the tissue structures in the chest X-ray greatly increases the complexity of the interpretation. For example, detection is challenging when the contrast between the lesion and the surrounding tissue is very low or when the lesion overlaps the ribs or large pulmonary blood vessels. Even for an experienced doctor, it is sometimes not easy to distinguish between similar lesions or to find very obscure nodules. Therefore, the examination of the lung disease in chest X-ray will cause a certain degree of missed detection. The wide application of chest X-rays and the complexity of reading them make computer-aided detection (CAD) systems a hot research topic since the system can help doctors to detect suspicious lesions that are easily missed, thus improving the accuracy of their detection.

The first attempt to establish a computer-aided detection system was in the 1960s [2], and studies have shown that the detection accuracy for the chest disease is improved with a X-ray CAD system as an assistant. Many commercial products have been developed for the clinical applications, including CAD4 TB, Riverain, and Delft imaging systems [3]. However, because of the complexity of the chest X-rays, the automatic detection of the diseases remains unresolved, and most of the existing CAD systems are aimed at the early detection of the lung cancer. A relatively small number of studies are devoted to the automatic detection of the other types of the pathologies [4].

The CAD systems are mainly divided into the following steps: image preprocessing, extracting ROI regions, extracting ROI features, and classifying disease according to the features. The recent development of artificial intelligence (AI) combined with the accumulation of large volumes of medical images opens up new opportunities for building CAD systems in the medical applications. Artificial intelligence methods (including shallow learning and deep learning, etc.), especially deep learning, mainly replace the process of feature extraction and disease classification in the traditional CAD systems. Artificial intelligence methods have also been widely used in image segmentation and bone suppression of chest X-ray. The shallow learning methods are widely used as classifiers to detect diseases, but their performance depends strongly on the extracted hand-crafted features. For the complex chest X-ray images, it takes a long time for researchers to find a good set of features that will be helpful of the CAD performance. Recently, due to the extensive and successful application of deep learning in different image recognition tasks (such as image classification [5–8] and semantic segmentation [9–12]), interest has been stimulated in reapplying deep learning to medical images. In particular, advances in deep learning and large database construction have made the algorithm “go beyond” the performance of medical professionals in a variety of medical imaging tasks, including pneumonia diagnosis [13], diabetic retinopathy detection [14], skin cancer classification [15], arrhythmia detection [16], and bleeding identification [17]. Therefore, deep learning methods (especially CNN), which automatically learn image features to classify chest diseases, have become a mainstream trend.

This article reviews the common methods of computer-aided detection of the chest radiographs based on AI. The second section provides commonly used CXRs datasets and general image preprocessing techniques applied to chest radiographs. The third section discusses the detection of single diseases, including tuberculosis, pulmonary nodules, interstitial lung disease and other diseases. The fourth section provides the detection of multiple diseases. The fifth section summarizes the CAD of chest radiography and discusses existing problems and development trends, finally, we conclude the paper in the sixth section.

## Datasets and image preprocessing techniques

### Datasets

**Fig. 1:**
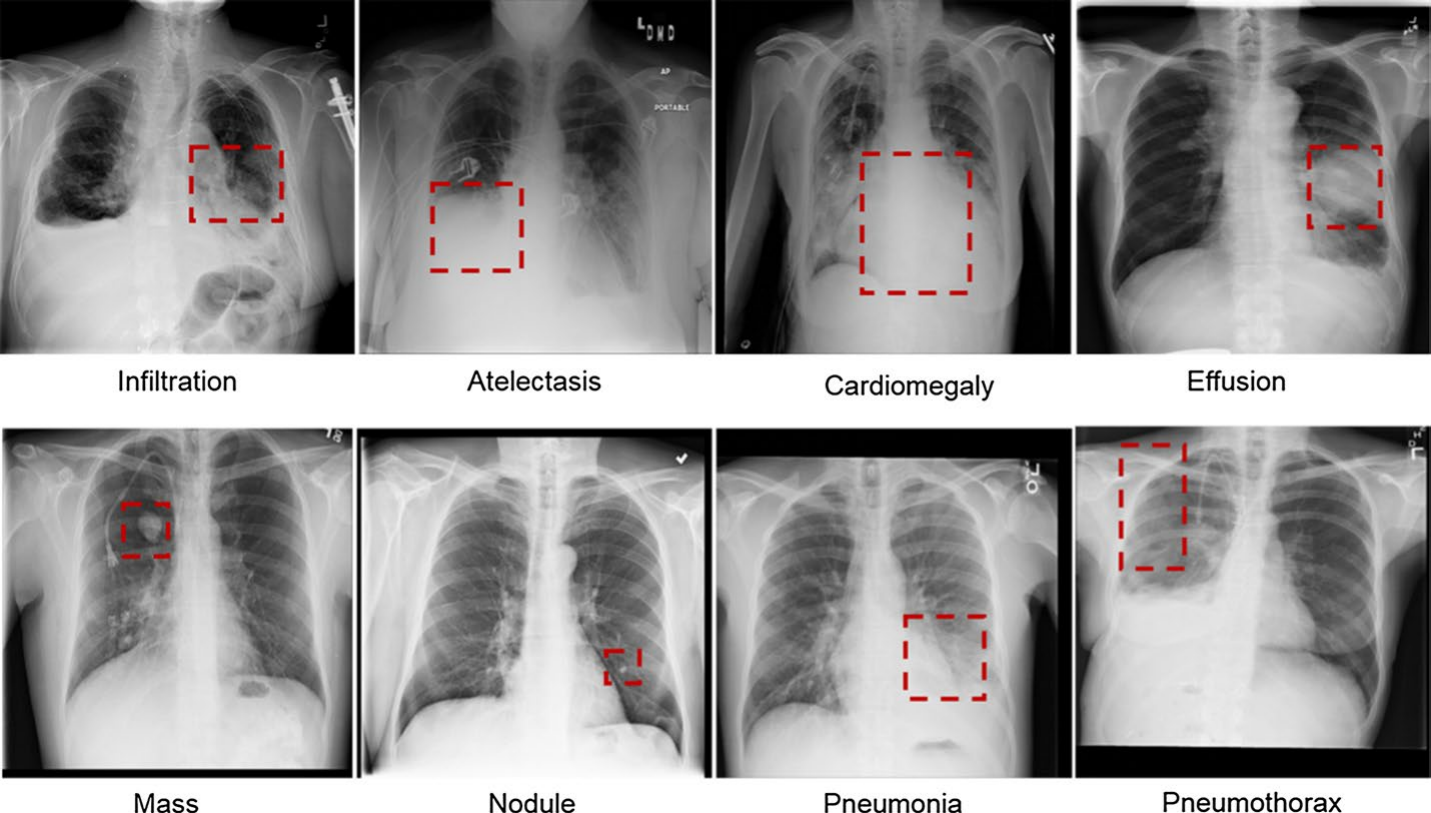
Eight common diseases such infiltration, atelectasis, cardiac hypertrophy, effusion, lumps, nodules, pneumonia, and pneumothorax observed in the chest radiographs

CAD systems can be used to detect various diseases in the chest X-rays. Figure 1 shows the eight most common types of diseases observed in the chest radiograph [23], which are the disease of infiltration, atelectasis, cardiac hypertrophy, effusion, lumps, nodules, pneumonia, and pneumothorax, respectively. The training, validation, testing, and performance comparisons of CAD systems require many chest radiographs. Since creating a large, annotated medical image dataset is not easy, most researchers rely on the following publicly available CXR datasets.

#### Indiana dataset [18]

The dataset was collected from various hospitals affiliated to the Indiana University School of Medicine. It consists of 7470 chest radiographs including the frontal and lateral images of disease annotations, such as cardiac hypertrophy, pulmonary edema, opacity, or pleural effusion.

#### KIT dataset [19]

The dataset consists of 10,848 DICOM cases from the Korea Tuberculosis Institute under the Korea Association of Tuberculosis, including 7020 cases of normal and 3828 cases of abnormalities (tuberculosis).

#### MC dataset [20]

The dataset was collected from the Department of Health and Human Services in partnership with Montgomery County, Maryland in the United States. The group consisted of 138 frontal chest radiographs from the Montgomery County Tuberculosis Screening Program, of which 80 were normal and 58 were tuberculosis with the image sizes as 4020 × 4892 or 4892 × 4020 pixels.

#### JSRT dataset [21, 22]

The dataset was compiled by the Japanese Society of Radiological Technology (JSRT) and includes 247 chest radiographs, of which 154 have pulmonary nodules (100 malignant and 54 benign), and 93 have no nodules. All of the X-ray images are 2048 × 2048 pixels in size, while the color depth of the grayscale is 12 bits.

#### Shenzhen dataset [20]

The dataset was collected in collaboration with Shenzhen No. 3 People’s Hospital, Guangdong Medical College, Shenzhen, China. It contains 662 cases of chest X-rays, including 326 normal cases and 336 tuberculosis cases.

#### Chest X-ray14 dataset [23]

The dataset is extracted from the clinical PACS databases in the hospitals affiliated to National Institutes of Health Clinical Center and consisted of about 60% of all frontal chest X-rays in the hospitals. The dataset contains the X-ray images of 112,120 frontal views of 30,805 patients and the image labels of 14 diseases (each image can have multiple labels) that can be mined from related radiology reports using natural language processing (NLP). The dataset contains 14 common chest pathologies, including atelectasis, consolidation, infiltration, pneumothorax, edema, emphysema, fibrosis, effusion, pneumonia, pleural thickening, cardiomegaly, nodule, mass, and hernia.

### Image preprocessing techniques

Computer-aided detection systems usually take input images for a series of preprocessing steps. The main purpose of preprocessing is to enhance the quality of the images and make the ROI (region of interest) more obvious. Thus, the quality of the preprocessing has a large influence on the performance of the subsequent procedures. Typical preprocessing techniques include image enhancement, image segmentation and bone suppression for specific applications in chest X-rays. This section briefly describes these techniques.

#### Enhancement

Contrast, edge features, and noise in images have a large influence on the classification and identification of lesions. To obtain more details in obscure and low-contrast areas of chest X-ray images, chest radiographs should be enhanced to highlight the structural information and suppress noise. The enhancement of chest X-rays includes contrast enhancement, noise suppression, edge sharpening, and filtering [24–27]. Contrast enhancement is the process of stretching the brightness value range in an image, which improves the overall or local contrast of the image and makes the image clear. Image sharpening compensates the contour of the image, enhances the edge of the image and the part of the grayscale jump, that is, enhances the image detail information. Noise suppression is the process of image denoising while preserving the details of the image as much as possible. In the image enhancement process, the filtering operation may be used, which can be carried out either in the real domain or in the frequency domain. Filtering is a neighborhood operator that uses the value of the pixels around a given pixel to determine the final output value of that pixel. In general, as a preprocessing step, the image enhancement can help reduce the rate of misdiagnosis without losing image details, introducing excessive noise and causing detail distortions.

#### Segmentation

In chest radiographs, it is usually necessary to segment the anatomy to obtain the ROI. Due to the different purpose (for detection of pulmonary nodules, cardiomegaly and abnormal asymmetry, etc.) of the tasks in chest radiography, there are many different studies focus on segmentation. Some studies segment the lung fields [28, 29]; others detect the contours of the lung fields [30] or ribs [31]; and a few try to directly detect the diaphragm or the costophrenic angle [32]. Lung field segmentation is the most important because it accurately defines the ROIs of the lung fields, where specific radiological signs, such as lung opacities, cavities, consolidation, and nodules, can be searched. Segmentation methods can be divided into image progressing-based methods and machine learning-based methods. Table 1 summarizes the segmentation methods which are evaluated by the used data, the assessment measures, and the corresponding segmentation results.

**Table 1:** Segmentation methods in chest X-ray. The datasets, methods, assessment measures, and segmentation results are provided in each column, respectively

	Study	Datasets	Assessment measures	Results
Image progressing based methods	Cheng et al. [34]	Custom	Accuracy	
	Armato et al. [35]	Custom (600)	Subjectively assessed the accuracy and completeness of the contour	Up to 79.1% (score 4 or 5) and 8.1% inaccurate (score 1 or 2)
	Li et al. [36]	Custom (40)	Accuracy, sensitivity, specificity	Left lung: 95.2% accuracy, 91% sensitivity, 96.5% specificity; right lung: 96% accuracy, 91.1% sensitivity, 97.2% specificity
	Iakovidis et al. [37]	Custom (24)	Accuracy, sensitivity, and specificity	95.3% sensitivity, 94.3% specificity
	Wan et al. [38]	JSRT Custom (154)	Accuracy, overlap scores, precision, sensitivity, specificity, and F score	Accuracy, F value, accuracy, sensitivity, and specificity were higher than 90%; the JSRT dataset overlap score was 87%; the overlap rate of the custom datasets (standard machines) was 81% and (mobile machines) is 69%
	Van Ginneken et al. [42]	Custom (230)	Overlap scores	Left lung: 0.887 ± 0.114; right lung: 0.929 ± 0.026
Machine learning based methods	Mcnittgray et al. [45]	Custom (33)	Accuracy	NN: 76%; LDA: 70%; KNN: 70%
	Vittitoe et al. [46]	Custom (198)	Sensitivity, specificity, and accuracy	Sensitivity: 0.907 ± 0.044; specificity: 0.972 ± 0.022; accuracy: 0.948 ± 0.016
	Shi et al. [47]	JSRT (52)	Accuracy	0.978 ± 0.0213
	Novikov et al. [51]	JSRT	Dice coefficient, jaccard coefficient	Lung: 97.4%, 95%; collarbone: 92.9%, 86.8%; heart: 93.7%, 88.2%
	Dai et al. [52]	JSRT, MC	Intersection-over-union	Both lungs: 94:7% ± 0:4%, heart: 86:6% ± 1:2%

Accuracy: (TP + TN)/(TP + TN + FP + FN); sensitivity: R = TP/(TP + FN); specificity: TN/(TN + FP); overlap scores: TP/(TP + FP + FN); precision (or positive predictive value): P = TP/(TP + FP); F score: 2 × P × R/(P + R); intersection-over-union: IoU = TP/(TP + FP + FN); negative precision: TN/(TN + FN); false accept rate: FAR = FP/(FP + TN); false rejection rate: FRR = FN/(TP + FN); where TP, TN, FP, and FN represent true positive, true negative, false positive, and false negative, respectively

Dice coefficient: DSC = 2 × (|S∩GT|)/(|S| + |GT|, jaccard coefficient of concordance: JS = (|S∩GT|)/(|S∪GT|), where S is segmentation result, GT is the ground truth

- Image progressing-based methods. The category can be subdivided into rule-based methods and deformable model-based methods. Rule-based algorithms segment the lung region using rules based on the location, intensity, texture, shape, and relationships with other anatomies [33], including thresholding, edge detection, region growth, mathematical morphology operations, geometric models matching methods, etc. [34–38]. Typical examples based on deformable model segmentation are the active shape model (ASM) [39], the active appearance model (AAM) [40], and improvements to both [41–44].
- Machine learning-based methods. The category can also be referred to as pixel-based methods. For chest radiographs, each pixel is assigned to a corresponding anatomical structure, such as lung, heart, mediastinum, diaphragm and so on. The classifier can use various features, such as the gray value of the pixel, spatial location information, and texture statistical information. There features are inputted into some classifier, e.g., a k-nearest neighbor (KNN) classifier, support vector machine (SVM), Markov random field (MRF) model, or neural network (NN), to train the classifier. The method can be subdivided into shallow machine learning-based methods and deep learning-based methods.
  - In shallow machine learning-based methods, the feature extraction process is intuitive, and the main challenge is to determine the appropriate categories of the features and extract them in a robust way. Mcnittgray et al. [45] first proposed a method of lung field segmentation using features. The features used were grayscale, a measure of the local difference, and a measure of the local texture. Using KNN, linear discriminant analysis (LDA), and feedforward backpropagation neural network (NN), the method classifies each pixel of a CXR into one of several anatomical categories (heart, sub diaphragm, upper mediastinum, lungs, armpit, and background). The correct percentages were 70%, 70%, and 76% for each of classifier, respectively. Similar to the literature [45], Vittitoe et al. [46] used spatial and texture information to segment CXRs into lungs or non-lungs. They used Markov Random Field to build a model that had high sensitivity, specificity, and accuracy. Shi et al. [47] used an unsupervised approach to segment the lung region in CXRs. They segmented lung fields using fuzzy C-means (FCM) clustering based on Gaussian kernels and space constraints. The method was tested on 52 CXRs of the JSRT dataset and achieved an accuracy of 0.978 ± 0.0213.
  - Shallow learning-based approaches rely on hand-crafted features that can become vulnerable when applied to different patient groups and image qualities. Because the traditional lung segmentation method requires human intervention and a priori knowledge of the dependence of the problems, the deep learning extractor has effectively replaced manual feature extraction. The current application of a more semantic segmentation method is the fully convolutional network (FCN) [48], which retains the advantageous features of SegNet [49], accepts input of any size, and produces output of the same size. Ronneberger et al. [50] improved the FCN to create a U-net structure consisting of a context-grabbing path and a symmetric extension path, allowing for precise positioning and reducing the number of images required for training. Subsequently, U-net was used for biomedical segmentation with good performance. For example, Novikov et al. [51] proposed a multiple image segmentation method based on U-net to segment the lung region and solve the data imbalance problem by associating the a priori class data distribution with a loss function. Additionally, this method goes beyond the advanced methods in the clavicle and heart segmentation tasks. Dai et al. [52] proposed a structure correcting adversarial network (SCAN) framework that uses a confrontational process to develop an accurate semantic segmentation model for segmenting lung fields and the heart in chest X-ray images. This method improves the FCN and achieves segmentation performance comparable to human experts.

#### Bone suppression

Bone suppression is a unique preprocessing technique in chest radiography and is an important preprocessing step in lung segmentation and feature extraction. The ribs and clavicle can block lung abnormalities, which complicates the feature extraction phase of a CAD system. Therefore, there is a need to remove skeletal structures, especially the posterior ribs and clavicle structures, to increase the visibility of the soft tissue density. Suzuki et al. [53] and Loog et al. [54] first proposed the bone suppression technique in 2006. Subsequent research has shown that using bone suppression techniques can improve the performance of pulmonary nodule detection [55–57] and can also be used to detect other abnormalities. For example, Li et al. [58] found that bone suppression could increase the performance on recognition of local pneumonia significantly.

A method of removing the skeletal structure of CXRs is mainly applied to dual-energy subtraction (DES) imaging [59]. DES radiography involves the use of X-ray radiation to take two radiographs at high energy and low energy. The two radiographs are then combined using a specific weighting factor to form a subtracted image that highlights soft tissue or skeletal components. However, the use of this technology requires specialized equipment, and only a few hospitals use the DES system.

A better solution is to automatically detect or remove bone structures in chest X-rays based on image processing techniques. Suzuki et al. [53] developed a method to suppress the contrast between ribs and clavicles in a chest X-ray with a multiresolution, large-scale training artificial neural network (MTANN). Subtracting a bone image from the corresponding chest radiograph produces a “soft tissue image”, where the rib and clavicle are substantially suppressed. Nguyen et al. [60] used independent component analysis (ICA) to separate the ribs and other parts of lung images. The results showed that 90% of the ribs could be completely and partially inhibited, and 85% of the cases increased the nodule visibility. Yang et al. [61] used deep convolution neural networks (ConvNets) as the basic prediction unit and proposed an effective deep learning method for single conventional CXR skeletal suppression. The results showed that this method can produce high-quality and high-resolution images of bone and soft tissue. Gordienko et al. [62] detected lung cancer using a deep learning method, which demonstrated the efficiency of the bone suppression technique. The study found that the pretreatment dataset without bones showed better accuracy and loss results.

## Specific disease detection

Chest X-rays contain the main respiratory and circulatory organs, maintaining some of the body’s vital life activities. Millions of people suffer from chest disease each year. Tuberculosis, interstitial lung disease (LID), pneumonia, lung cancer, and other diseases are the most common diseases in the world [4]. In chest radiographs, there are three main types of anomalies: texture abnormalities, which are characterized by diffuse changes in the appearance and structure of the area, such as interstitial lesions; focal abnormalities, which are manifested as isolated changes in density, such as pulmonary nodules; and abnormal shape, in which disease processes change the outline of the normal anatomy, such as cardiomegaly. Sometimes, the texture and shape of the chest changes at the same time as a certain disease, such as tuberculosis [63]. The section describes common abnormalities in chest radiographs mainly caused by pulmonary nodules, tuberculosis, interstitial lesions, cardiomegaly, etc.

### Pulmonary nodule detection

According to the World Cancer Report, lung cancer is the most common cancer in men and the third most common in women. It is one of the most aggressive human cancers, with a 5-year overall survival of 10–15% [64]. Pulmonary nodules are early manifestations of lung cancer; thus, the early detection and diagnosis of pulmonary nodules is very important for the early diagnosis and treatment of lung cancer. Nodules often appear on chest radiographs as small circular or oval low-contrast tissue masses in the lung region. They are characterized by several features, e.g., large changes in size, large changes in density, uncertainty of location in the lung area, etc. [65]. Creating a lung nodules automatic detection algorithm has always been a difficult but important aspect in the field of medical image CAD. Table 2 summarizes the main methods of pulmonary nodule detection, assessment measures, and results.

**Table 2:** Pulmonary nodule detection. The datasets, assessment measures, and detection results are provided in each column, respectively

Study	Datasets	Assessment measures	Results
Wei et al. [70]	JSRT	AUC	85%
Schiham et al. [67]	JSRT	Average sensitivity under FP/image	2 FP/image: 51%; 4 FP/image: 67%
Shiraishi et al. [71]	Custom (924)	Average sensitivity under FP/image	5.05 FP/image: 70.1%;
Chen et al. [65]	JSRT Custom (48)	Average sensitivity under FP/image	5 FP/image: JSRT, 78.6%; Custom, 83.3% 2 FP/image: JSRT, 71.4%; Custom, 77.1%
Hardie et al. [72]	Custom (167) JSRT	Average sensitivity under FP/image	4 FP/image: sensitivity 78.1%
Ogul et al. [73]	JSRT Custom (300)	Average sensitivity under FP/image	JSRT: 6.4 FP/image, 80% Custom: 6.7 FP/image, 76%
Bush. [76]	JSRT	Sensitivity and specificity	Sensitivity: 92%; specificity: 86%
Wang et al. [77]	JSRT	Average sensitivity and specificity under FP/image	1.19 FP/image: sensitivity 69.27%; specificity 96.02%;

FP/image means specific false positives per image. AUC denotes area under the receiver operating characteristic curve

To prove that CAD is clinically useful for radiologists in detecting pulmonary nodules on chest radiographs, Kobayashi et al. [66] conducted observer performance studies. In this trial, 60 cases of chest radiographs that contained pulmonary nodules and 60 cases of non-nodular chest radiographs were used. The 16 radiologists who participated in the trial explained chest radiographs without computer-aided and with computer-aided interventions. Radiologists were evaluated for the performance of distinguishing the lung nodule using receiver operating characteristic curves (ROCs). The results showed that CAD systems increased the accuracy of the radiologist’s detection of pulmonary nodules from 0.894 to 0.940.

Traditional pulmonary nodule CAD systems include image preprocessing (enhancement and lung segmentation), candidate nodule detection, and extraction of features to reduce false positives [4, 67]. The purpose of image preprocessing is to enhance nodules, partition lung tissue, remove other tissue areas, and reduce data noise. Candidate nodule detection uses a variety of algorithms to identify as many of the nodules in the image as possible. To enhance the sensitivity of the algorithm to the nodules, this step does not strictly require a false alarm rate. False positive reduction in the suspected concentration removes the non-nodules and reduces the false positive false alarm rate of the system. At present, many algorithms focus on how to improve the detection rate of the nodules while reducing the false positives in the detection results.

To reduce the false positive nodules, the traditional algorithm extracts the features of the candidate nodules and classifies the nodules and non-nodules by the features. These features have a significant impact on the CAD performance [68, 69]. Early studies of lung nodule detection used differences in the candidate shapes at different thresholds as a feature to identify the candidate nodules. However, these methods consider only the intensity and shape of the lung nodule candidates, and they cannot achieve high sensitivity and low false positive rates. Recent studies on pulmonary nodule detection have added gradient features (including intensity and direction) and texture features to identify lung nodules from pre-detected candidates. For example, Wei et al. [70] determined the optimal feature set of 210 features using the forward-step selection method. This method achieved good lung nodule sensitivity. However, with too many features, robustness cannot be guaranteed. Schiham et al. [67] used a multiscale Gaussian filter to extract 96 texture features as well as two location features and 11 lung nodule characterization detector features and classified them using the KNN classifier. Shiraishi et al. [71] extracted 57 image features from original and nodule-enhanced images based on the geometry, grayscale, background texture, and edge gradient features. Fourteen image features were extracted from the corresponding locations in the subtracted images, and three consecutive artificial neural networks (ANNs) were used to reduce the number of false positive candidates. Chen et al. [65] enhanced the nodules in images and used a clustering watershed algorithm to extract the initial candidate nodules. Thirty-one features, including morphology, gray intensity, area, and gradient, were extracted to identify pulmonary nodules using a nonlinear SVM with a Gaussian kernel. Hardie et al. [72] calculated a set of 114 features for each candidate nodule. The final test was performed on a subset of 46 features using the Fisher linear discriminant (FLD) classifier. Ogul et al. [73] used supervised methods to distinguish nodules and non-nodules via a set of representative image features. However, inaccurate feature calculations and segmentation of complex objects can introduce new errors in their performance because hand-crafted features do not adequately represent the nodules; moreover, the design of these features requires specialized prior knowledge.

With the development of convolution neural network (CNN) in recent years, CNN model has proved its performance in image classification and detection. However, the chest radiograph datasets used for lung nodule detection are relatively small, making it might not be very successful to train a complicated pulmonary nodule image neural network from scratch. To accomplish this goal, the following studies explored and used transfer learning [74]. Transfer learning is considered to be an efficient learning technique, especially when faced with relatively limited medical datasets. Compared with the “starting from scratch” way of most other learning models, transfer learning helps train new models by transferring the learned model parameters trained on a large datasets to the new model. Considering that some data or tasks are related, we can share the model parameters (also known as the knowledge) learned from the model into the new model to improve the model performance. Concretely, in the detection of chest X-ray diseases, it is to learn general semantic features (such as edge information, color information, etc.) from classification tasks (such as natural image classification) related to disease detection to improve the generalization of disease detection. The network learns the advanced semantic classification features by self-adjusting on the chest dataset to achieve the purpose of distinguishing specific types of diseases. Bar et al. [75] explored the feasibility of training a CNN with ImageNet, a well-known large scale non-medical image database, and finally the trained CNN model was applied to distinguish the diseases in chest radiograph. The best performance was achieved using a combination of features extracted from the CNN and a set of low-level features including: SIFT, GIST, PHOG and SSIM. This is the first-of-its-kind experiment that shows that deep learning with large scale non-medical image databases may be sufficient for general medical image recognition tasks. Based on this approach, Bush [76] explored the use of the RESNET CNN model by transfer learning the pre-training weights extracted from the ImageNet to classify pulmonary nodules, with a sensitivity of 92% and a specificity of 86%. The model can determine the general nodular area but cannot determine the exact locations of the nodules. Although advanced features can be derived from classical deep learning models that used transfer learning, they are not related to medical image analysis tasks. The greater the gap between features extracted from natural images and those from medical images implies lower transferability of the feature. Wang et al. [77] fused the deep feature obtained from the CNN model used transfer learning and hand-crafted features (geometric features, intensity and contrast features, etc.) to reduce the false positive results. With the guidance of the specific false positives, i.e., 1.19 false positives per image, the sensitivity is 69.27% and the specificity is 96.02%. The low sensitivity is likely due to hand-crafted features being not superior.

### Tuberculosis detection

Tuberculosis (TB) is the ninth leading cause of death worldwide and the leading cause from a single infectious agent, ranking above HIV/AIDS. Globally, in 2016, the proportion of people who developed TB and died from the disease (case fatality ratio, CFR) was 16%, which meant that an estimated 10.4 million people (90% adults; 65% male; 10% people living with HIV) fell ill with TB [78]. Detecting tuberculosis in CXRs is a difficult task since it has different manifestations. Abnormal tuberculosis manifestations often affect the lungs’ texture and geometry in CXRs, such as consolidations, infiltrates and cavitation, ranging from subtle miliary patterns to obvious effusions [63, 79]. Table 3 summarizes the methods of tuberculosis detection, assessment measures, and results.

**Table 3:** Tuberculosis detection. The datasets, manifestations, assessment measures and results are shown in each column, respectively

Study	Datasets	Manifestations	Assessment measures	Results
Rohmah et al. [80]	Custom (120)	Tuberculosis	Accuracy, false accept rate, false rejection rate	95.7%, 3.33%, 6.67%
Tan et al. [81]	Custom (95)	Tuberculosis	Accuracy, sensitivity, specificity, AUC, precision	92.9%, 91%, 95.4%, 92.8%, 94.9%
Noor et al. [82]	TPR (100)	Tuberculosis	Accuracy	94%
Song et al. [87]	Custom (200)	Focal opacities	Accuracy	
Shen et al. [88]	Custom (243)	Cavities	True positive rate (or sensitivity) under FP/image	0.237 FP/image: 82.35%
Xu et al. [89]	Custom	Cavities	Densitivity, specificity, and accuracy	E-Group: 78.8%, 86.8%, 82.8%; D-Group: 69.4%, 81.6%, 75.5%
Hwang et al. [90]	KIT, MC, Shenzhen	Tuberculosis	AUC, accuracy, positive precision, negative precision	96.4%, 90.3%, 95.3%, 97.4%
Lakhani et al. [91]	Shenzhen	Tuberculosis	AUC, sensitivity, and specificity	99%, 97.3%, 100%

Some studies detected tuberculosis based on the shape, texture, and local characteristics of the lungs, focusing on the general performance. To imitate radiologists for visual detection and diagnosis of the texture features of chest X-ray images, Rohmah et al. [80] used texture features as descriptors to classify images as tuberculosis or non-tuberculosis. The results showed that tuberculosis can be detected based on the statistical features in the image histogram. Tan et al. [81] proposed a tuberculosis index (TI) based on the segmented pulmonary regional texture features and classified the normal and abnormal CXR using a decision tree, and obtained an accuracy rate of 94.9%. Noor et al. [82] proposed a statistical interpretation technique to detect tuberculosis in CXR images. They first applied the wavelet transform to the CXR image, calculated 12 texture measures from the wavelet coefficients, reduced the dimensions with PCA, and estimated the probability of misclassification using the probability ellipsoid and discriminant functions. In addition to extracting texture features, some studies applied bone suppression to pretreat chest radiographs for improving the classification performance. Leibstein et al. [83] used a DES rib block to pretreat chest radiographs and proposed a method based on the local binary pattern (LBP) and Laplacian of Gaussian (LoG) to detect tuberculosis and improve the classification performance. Maduskar et al. [84] compared the automatic tuberculosis detection of conventional CXRs and bone suppression CXRs and found that bone suppression was better than the conventional classification performance due to the diversity of pulmonary tuberculosis manifestations in the chest. Hogeweg et al. [85] improved the detection performance with help of an anomaly detection system and normal anatomy. The combination of texture anomaly detection and clavicle detection reduced false positives. In a study [86], the authors fused the supervisory subsystems for detecting the texture, shape, and focal abnormalities and developed a generic framework for tuberculosis detection.

Another portion of the literature has focused on the detection of specific manifestations, such as diffuse opacity, effusion, cavities, and nodule lesions. Song et al. [87] proposed a method to locate focal opacities in tuberculosis. These investigators studied the initial extraction of rib threads. After locating the ribs, morphological opening operations and seed growth methods were used to automatically locate the focal opacity. However, handling images that have blots or have no visible features on the border is not sufficient and can even lead to misjudgment. Shen et al. [88] proposed a Bayesian classification method based on hybrid knowledge to automatically detect tuberculosis cavities in CXRs. The gradient inverse coefficient of variation (GICOV) describes the texture (area boundary), and the circular measure describes the shape of the latent cavity. This method is the first automatic algorithm that detects tuberculosis accurately but uses a global adaptive threshold in such a way that automatic initialization cannot place the initial contour within the cavity, leaving a cavity. Xu et al. [89] classified tuberculosis cavities by combining texture and geometric features. First, rough feature classification was performed using Gaussian model-based template matching (GTM), LBP, and directional gradient histogram (HOG) methods to extract cavity candidates from CXR images. These candidates were then further refined using Hessian matrix eigenvalues and snake-based techniques by means of active contouring. In the final phase, SVM was used to reduce the false positives by further narrowing the enhanced cavity candidates at finer scales.

Most prior CAD algorithms used well-designed morphological features to distinguish different types of lesions and to improve the screening performance. However, such manual features do not guarantee the best description of tuberculosis classification. Recently, the role of deep learning in tuberculosis classification has proven to be effective. Hwang et al. [90] proposed the first CNN-based automatic tuberculosis detection system. To overcome the difficulty of training deep NNs, the author adopted a transfer learning strategy to improve the system’s performance. Lakhani et al. [91] used radiologist-enhanced methods to further improve the accuracy in cases of ambiguous classification, and they obtained an AUC of 0.99.

### Interstitial lung disease detection

The interstitial lung is support tissue outside the alveolar and terminal airway epithelium. When the interstitial lung is damaged, the chest radiograph indicates changes in the texture of the lung [92], such as linear, reticular, nodular, honeycomb, etc. [93]. Interstitial lung disease (ILD) is a group of basic pathological lesions with diffuse pulmonary parenchyma, alveolar inflammation, and interstitial fibrosis, including interstitial pulmonary edema, allergic pneumonia, idiopathic pulmonary interstitial fibrosis, sarcoidosis, and lung lymphatic cancer [94, 95]. Cases with different interstitial lesions behave very similarly on light sheets, even for professionals, it is difficult to distinguish between normal and non-normal tissue based on texture. Therefore, the detection of ILD in chest radiography is one of the most difficult tasks for radiologists.

Earlier articles used CAD systems to detect ILD in chest radiographs through texture analysis [96–99]. For example, the CAD system of the Kun Rossman Laboratory in Chicago [100] divided the lung into multiple regions of interest and analyzed the lungs’ ROI to determine whether there was any abnormalities. Then, pretrained NNs were used to classify suspicious areas to be detected. This system can help doctors improve the accuracy of interstitial lesion detection.

Plankis et al. [94] developed a flexible scheme for CAD of ILD. This approach can detect a variety of pathological features of interstitial lung tissue based on an active contour algorithm which can select the lung region. The region is then divided into 40 different regions of interest. Then, a two-dimensional Daubechies wavelet transform is performed on the ROI to calculate the texture measure. However, with the extensive application of deep learning in the detection of lung diseases, there is little literature on the detection of interstitial lung disease in the absence of a large chest X-ray dataset on ILD. Most of the literature used CT datasets to detect ILD.

### Other diseases

In chest X-rays, in addition to pulmonary nodules, tuberculosis, and ILD, there are other diseases that can be detected, such as cardiomegaly, pneumonia, pulmonary edema, and emphysema. There is less literature on these diseases, and a brief discussion is given here.

Detecting cardiomegaly usually requires analyzing the heart size and calculating the cardiothoracic ratio (CTR) and developing a cardiac tumor screening system. Candemir et al. [30] used 1D-CTR, 2D-CTR, and CTAR as features, and they used SVM to classify 250 cardiomegaly images and 250 normal images, obtaining an accuracy of 76.5%. Islam et al. [101] used multiple CNNs to detect cardiomegaly. The network was accurately adjusted on 560 image samples and validated on 100 images, and they obtained a maximum accuracy of 93%, which is 17% points higher than in the literature [30].

Pneumonia and pulmonary edema can be classified by extracting texture features. Parveen et al. [102] used an FCM clustering algorithm to detect pneumonia. The results showed that the lung area of the chest was low in black or dark gray. When a patient has pneumonia, the lungs are full of water or sputum. Thus, there will be more absorbed radiation, and the lung areas will be white or light gray. This approach can help doctors detect the degree of infection easily and accurately. Kumar et al. [103] used a machine learning algorithm to perform texture analysis of chest X-rays. They used a Gabor filter and SVM to distinguish normal chest and pulmonary edema in chest radiographs, and they obtained an AUC of 0.96. Here, they did not use large datasets and validate other lung conditions. Islam et al. [101] used a CNN to detect and locate pulmonary edema, which manifested as a reticular white structure in the lung area with no anatomical changes.

## Multiple disease detection

There may be one or more diseases in the chest radiographs. However, when using CAD methods to output the normal/abnormal classification of each image, i.e., when they are applied to only detect one disease, this approach cannot meet the demand. This section discusses the methods of using CAD technique to detect multiple diseases. Table 4 summarizes the conditions, methods, assessment measures, and results.

**Table 4:** Multiple disease detection. The datasets, manifestations, assessment measures and results are shown in each column, respectively

Study	Datasets	Conditions	Measurements	Results
Avni et al. [104]	Custom	Left and right pulmonary pleural effusion, cardiomegaly, and septum enlargement	AUC	Left and right pulmonary pleural effusion: 80%; cardiomegaly: 79.2%; septum enlargement: 88.2%
Noor et al. [105]	Custom	Lobar pneumonia, tuberculosis, and lung cancer	Accuracy	70%, 97%, and 79%, respectively
Bar et al. [75]	Custom	Right pleural effusion, cardiomegaly, health, and abnormal disease	AUC	93%, 89%, and 79%, respectively
Cicero et al. [106]	Custom	Normal, cardiomegaly, pleural effusion, pulmonary edema, and pneumothorax	AUC	96.4%, 87.5%, 85%, 96.2%, 86.8%, and 86.1%, respectively
Wang et al. [23]	Chest-Xray14	14 common diseases in CXRs	AUC	Mean: 73.8%
Yao et al. [107]	Chest-Xray14	14 common diseases in CXRs	AUC	Mean: 80.3%; however, limited training focuses on biased interdependence and cannot accurately represent the actual distribution of morbidities
Rajpurkar et al. [13]	Chest-Xray14	14 common diseases in CXRs	AUC	Mean: 84.2%; pneumonia (76.8%) exceeded the human level
Kumar et al. [109]	Chest-Xray14	14 common diseases in CXRs	AUC	Mean: 79.5%; cardiomegaly (91.33%) beyond the previous method
Guan et al. [111]	Chest-Xray14	14 common diseases in CXRs	AUC	Mean: 87.1%

Avni et al. [104] used the “bag of visual words” to represent the image content and used a nonlinear, multiple SVM to classify the left and right pulmonary pleural effusion, cardiomegaly, and septum enlargement, and they obtained AUCs of 80%, 79.2%, and 88.2%, respectively. However, their algorithm was designed only for global representation and could identify the diseases manifested in the locally or relatively small regions.

Noor et al. [105] proposed a new texture-based statistical method for the detection of lobar pneumonia, tuberculosis, and lung cancer. Each ROI was transformed into four subsets using a two-dimensional Daubechies wavelet transform, which represented the trend, horizontal, vertical, and diagonal detail coefficients. Twelve types of texture measurements, such as the mean energy, entropy, contrast, and maximum column total energy, were calculated. The modified principal component (ModPC) method was used to generate the feature vectors for the discrimination process. For all three diseases, the texture measurement of maximum column total energy produced a 98% correct classification rate. The two diseases were then compared in pairs, and the correct classification rates for lobar, tuberculosis, and lung cancer using mean energy and maximum texture measurements were 70%, 97%, and 79%, respectively. This algorithm is different from other semi-automatic methods in that the ROI choice does not involve the usual segmentation problem, and the proposed statistical-based CAD algorithm does not rely on establishing precise boundaries and avoids the possibility of losing information from the original image. However, this method still requires further work that involves larger samples for validation studies.

Bar et al. [75] used a combination of features extracted from a CNN, which were trained on ImageNet, and a set of low-level features to detect right pleural effusion, cardiomegaly, and health versus abnormal disease; they obtained AUC values of 93%, 89%, and 79%, respectively. However, the detection was performed with features learned from non-medical datasets, and it was inaccurate.

Cicero et al. [106] used the GoogLeNet CNN to classify normal, cardiomegaly, pleural effusion, pulmonary edema, and pneumothorax on a moderate size dataset automatically. It was shown that the current CNN architecture can be trained with a medical dataset of moderate size, which solves the problem of simultaneous prediction of multiple labels for the detection and removal of common diseases in chest radiographs.

NIH [23] published a large-scale chest X-ray dataset and used a weak-supervised multi-label method to classify and locate eight diseases, which validated the usability of deep learning on this dataset. Based on this result, Yao et al. [107] used DENSENET to extract disease features on this dataset and proposed LSTM-based approach to simulate label dependency, which improved the classification performance. Rajpurkar et al. [13] proposed the ChexNet method, which used dense connections [108] and batch normalization [109] to make the optimization of such a deep network tractable. The AUC of pneumonia on the Chest-Xray14 dataset was 76.4%, reaching the human level, and greatly improved the accuracy of detection of these 14 diseases. Kumar et al. [110] used a cascade deep learning network to classify 14 diseases on this dataset, and the performance of classification of cardiomegaly improved upon previous methods. These methods of detecting multiple labels in chest radiographs all input the global image into the network. However, the lesion area can be very small compared to the global image, and using a global image for classification could result in a significant amount of noise outside the lesion area. In response to this problem, Guan et al. [111] proposed the attention guided convolutional neural network (AG-CNN), which has three branches, i.e., global branch, local branch, and fusion branch. This network combines global and local information to improve the recognition performance.

## Discussion

This study discussed various CAD algorithms for detecting abnormal chest radiographs. CXR CAD algorithms have wide applications in detecting various diseases, and they are playing a vital role as a second opinion for medical experts. In addition, CAD algorithms also reduce the workload of medical experts by reviewing many CXRs quickly. In this section, we will discuss and compare the CAD algorithms in detection of chest abnormalities, and will state some problems and the future works in this field.

1. From the literatures, it can be found that there are many CAD methods currently used to detect abnormalities in chest radiographs. Most of these methods belong to the field of artificial intelligence [112] and they dedicate to computer-aid detection based on the chest radiograph. However, it is proved that the deep learning methods are more accurate in classification from the comparison and laboratory experiments shown in Table 5. Furthermore, previous methods can only detect one or several diseases from the chest radiograph, while Table 6 shows that the deep learning method can read the appearance of various types of suspected diseases simultaneously and directly from the chest radiograph, which conforms to the principles of radiologists’ interpretation of the film. This should be a main direction of computer-assisted detection of chest radiographs in the future. We think that deep learning has very far-reaching development potential, while at the same time it needs further improvement for playing a greater role. At present, deep learning methods classify diseases by extracting the features from the limited datasets. However, the problems with these methods are that limited datasets have limitations, such as unbalanced sample distribution, and the generality of the networks trained with them is insufficient. In order to further improve the classification ability, the following aspects should be carried out: (1) Dataset: Based on existing datasets, a more representative dataset with a larger number of examples, preferably from different devices and different regions, is established, making the training network more versatile. (2) The network needs to be further studied and optimized in order to facing special resolution. For example, RESNET increased network depth by adding residual blocks [7]. DENSENET connected all layers (with matching feature-map sizes) directly with each other to ensure maximum information flow between layers in the network. They can farther alleviate the vanishing-gradient problem, strengthen feature propagation, encourage feature reuse, and substantially reduce the number of parameters [108]. Dual Path Network [113] combined residual channels with densely connected paths to increase training speed significantly, reduce memory footprint, and maintain higher accuracy. Similar to the literature [111], the Residual Attention Network [114] introduced an attention mechanism to extract the significant features from the images by stacking multiple attention modules. These mentioned above methods have greatly improved the classification accuracy.

**Table 5:** Comparison of classification methods for thoracic diseases. The classification methods, measurements, and best results in the review are shown in each column, respectively

	Methods	Measurements	Best results
Traditional machine learning methods	Maharanobis distance [70]	AUC	Lung nodules: 85%
	KNN [67]	Sensitivity	Lung nodules 4FP/image: 67%
	ANN [71]	Sensitivity	Lung nodules 5.05FP/image: 70.1%
	SVM [30, 65, 89, 104]	Sensitivity, accuracy Specificity, AUC	Lung nodules: sensitivity 5FP/image: 83.3% Tuberculosis: accuracy 82.8%, specificity 86.8%, sensitivity 78.8% Cardiomegaly: accuracy 76.5%, sensitivity 77.1%, AUC 79.2% Pleural effusion: AUC 80% Septum enlargement: AUC 88.2%
	Fisher linear discriminant [72]	Sensitivity	Lung nodules 4FP/image: 78.1%
	Minimum distance [80]	Accuracy	Tuberculosis: 95.7%
	Decision tree [81]	Accuracy	Tuberculosis: 94.9%
	Bayesian classifier [88]	Sensitivity	Tuberculosis: 0.237 FP/image: 82.35%
Traditional machine learning methods + CNN	CNN transfer learning + SVM [75]	AUC	Right pleural effusion: 93% Cardiomegaly: 89%
	AlEXNET transfer learning + random forests [77]	Sensitivity, specificity	Lung nodules: 1.19FP/image: sensitivity 69.27%, specificity 96.02%
Deep learning methods	RESNET transfer learning [76]	Sensitivity Specificity	Lung nodules: sensitivity 92%, specificity 86%
	CNN transfer learning [90]	AUC, accuracy	Tuberculosis: 96.4%, 90.3%
	CNN [101]	Sensitivity, accuracy, AUC, specificity	Cardiomegaly: 93%, 97%, 94%, 92%
	GoogleNet CNN [106]	AUC	Cardiomegaly: 87.5%, pneumothorax: 86.1%, pleural effusion: 96.2%, pulmonary edema: 86.8%

**Table 6:** Comparison of multiple label classification methods for thoracic diseases. The classification methods, measurements, and best results in the review are shown in each column, respectively

Methods	Thoracic diseases	Measurements	Best results
RESNET [23]	Atelectasis, cardiomegaly effusion, infiltration, mass, nodule, pneumonia, pneumothorax, consolidation, edema, emphysema, fibrosis, pleural thickening hernia	AUC	Respectively, 71.6%, 80.7%, 78.4%, 60.9%, 70.6%, 67.1%, 63.3%, 80.6%, 70.8%, 83.5%, 81.5%, 76.9%, 70.8%, 76.7%
LSTM + DENSENET [107]		AUC	Respectively, 77.2%, 90.4%, 85.9%, 69.5%, 79.2%, 71.7%, 71.3%, 84.1%, 78.8%, 88.2%, 82.9%, 76.7%, 76.5%, 91.4%
ChexNet [13]		AUC	Respectively, 82.1%, 90.5%, 88.3%, 72.0%, 86.2%, 77.7%, 76.3%, 89.3%, 79.4%, 89.3%, 92.6%, 80.4%, 81.4%, 93.9%
Cascade deep learning network based on DENSENET [110]		AUC	Respectively, 76.2%, 91.3%, 86.4%, 69.2%, 78.9%, 70.4%, 71.5%, 85.9%, 78.4%, 88.8%, 91.6%, 75.6%, 77.4%, 89.8%
Attention guided CNN [111]		AUC	Respectively, 85.3%, 93.9%, 90.3%, 75.4%, 90.2%, 82.8%, 77.4%, 92.1%, 84.2%, 92.4%, 93.2%, 86.4%, 83.7%, 92.1%
2. The big data is used in current deep learning methods to extract the features of the corresponding disease through the convolution algorithm, that is, to extract different features from shallow level to deep level through convolution operations. The training process of the neural network makes the entire network to automatically adjust the parameters of the convolution kernel, resulting in the suitable classification features being consistent with the appearance of the chest radiograph image. Although these methods have made great progress in this area, it is very time-consuming to build big data. Therefore, it should be considered whether future studies can use other domain datasets to emulate chest radiographs. Or similar to Two-Pathway Generative Adversarial Network (TP-GAN) proposed by literature [115] (combining a realistic frontal face view by simultaneously sensing the global structure and local details), it may be possible to use small datasets to generate valuable big data that can help with lung diseases detection. To avoid the need for large datasets, a method similar to Alphago Zero [116], which uses no datasets and instead relies on radiologist-interpreting-film-rules studied in the review, could be used. This should be a direction of study in the future.
3. According to the first point mentioned above, CNN and other networks have reached a relatively mature stage and have higher classification accuracy than other artificial intelligence methods. However, it should be believed that in the future research, in addition to the above-mentioned improvements on deep learning network, some traditional machine learning methods still have potential for development and will continue to play a vital role in many aspects. It is valuable to study these methods for improvement of chest X-ray disease detection.
4. The appearance of a disease in the chest is usually accompanied by other diseases and is related to each other, such as pulmonary tuberculosis usually accompanied by pneumonia, and there are other abnormalities that can be caused by the exacerbation of a disease. According to the multiple diseases detection section, multiple disease detection through artificial intelligence methods in the chest radiographs is clinically required, and is currently an important study direction. We can also consider the further detection of the deterioration of the disease, such as whether ordinary pneumonia can be transformed into interstitial pneumonia. The current methods do not reach this point, because the features of diseases that could further deteriorate have not been discovered yet. It could be solved by using CAD techniques in molecular biology, which plays an important role in the diagnosis and treatment of diseases.

## Conclusions

Usually there are four steps in a CAD system: algorithm preprocessing, extracting ROI regions, extracting ROI features, and classifying disease according to the features. In the algorithm preprocessing and extraction of ROI, the techniques of enhancement and segmentation are very important. Usually, there are many ways to highlight lesions and suppress noise. In the segmentation, the deformable model and the deep learning method are the best, while the rule-based methods have poor performance, and they often used together with other methods to improve the segmentation performance. The techniques of bone suppression are used less frequently in the literature, but removing the rib and clavicle that block lung abnormalities can improve the system performance; In terms of feature extraction, the features extracted by traditional machine learning algorithms include geometric features, texture features, and shape features, which are usually processed to reduce the dimensionality due to feature redundancy. However, hand-crafted features could have errors that affect the classification performance and are gradually replaced by deep learning methods. In terms of classifier selection, the performance of support vector machine and random forest in traditional algorithms may be better, but with the excellent performance of deep learning in image classification, the deep learning methods have gradually become the mainstream.

## References

[1] X-ray (radiography)—chest. https://www.radiologyinfo.org/en/info.cfm?pg=chestrad. Accessed 10 June 2018.

[2] Lodwick GS, Keats TE, Dorst JP (1963). The coding of roentgen images for computer analysis as applied to lung cancer. Radiology.

[3] Zakirov AN, Kuleev RF, Timoshenko AS, Vladimirov AV (2015). Advanced approaches to computer-aided detection of thoracic diseases on chest X-rays. Appl Math Sci.

[4] van Ginneken B, Hogeweg L, Prokop M (2009). Computer-aided diagnosis in chest radiography: beyond nodules. Eur J Radiol.

[5] Krizhevsky A, Sutskever I, Hinton GE (2017). ImageNet classification with deep convolutional neural networks. Commun ACM.

[6] Simonyan K, Zisserman A. Very deep convolutional networks for large-scale image recognition. arXiv preprint arXiv:14091556. 2014. http://arxiv.org/abs/1409.1556v6.

[7] He K, Zhang X, Ren S, Sun J. Deep residual learning for image recognition. In: 2016 IEEE conference on computer vision and pattern recognition (CVPR). Las Vegas, NV, USA: IEEE. 2016. p. 770–8. 10.1109/CVPR.2016.90.

[8] Szegedy C, Liu W, Jia Y, Sermanet P, Reed S, Anguelov D, et al. Going deeper with convolutions. In: 2015 IEEE conference on computer vision and pattern recognition (CVPR). Boston, MA, USA: IEEE. 2015. p. 1–9. 10.1109/CVPR.2015.7298594.

[9] Long J, Shelhamer E, Darrell T. Fully convolutional networks for semantic segmentation. In: 2015 IEEE conference on computer vision and pattern recognition (CVPR). Boston, MA, USA: IEEE. 2015. p. 3431–40. 10.1109/CVPR.2015.7298965. 27244717

[10] Mostajabi M, Yadollahpour P, Shakhnarovich G. Feedforward semantic segmentation with zoom-out features. In: 2015 IEEE conference on computer vision and pattern recognition (CVPR). Boston, MA, USA: IEEE. 2015. p. 3376–85. 10.1109/cvpr.2015.7298959.

[11] Noh H, Hong S, Han B. Learning deconvolution network for semantic segmentation. In: IEEE I conf comp vis. Santiago, Chile: IEEE. 2015. p. 1520–8. 10.1109/iccv.2015.178.

[12] Chen L, Papandreou G, Kokkinos I, Murphy K, Yuille AL (2018). Semantic image segmentation with deep convolutional nets and fully connected CRFs. IEEE Trans Pattern Anal Mach Intell.

[13] Rajpurkar P, Irvin J, Zhu K, Yang B, Mehta H, Duan T, et al. CheXNet: radiologist-level pneumonia detection on chest x-rays with deep learning. arXiv preprint arXiv:171105225. 2017.

[14] Gulshan V, Peng L, Coram M, Stumpe MC, Wu D, Narayanaswamy A (2016). Development and validation of a deep learning algorithm for detection of diabetic retinopathy in retinal fundus photographs. JAMA.

[15] Esteva A, Kuprel B, Novoa RA, Ko J, Swetter SM, Blau HM (2017). Dermatologist-level classification of skin cancer with deep neural networks. Nature.

[16] Rajpurkar P, Hannun AY, Haghpanahi M, Bourn C, Ng AY. Cardiologist-level arrhythmia detection with convolutional neural networks. arXiv preprint arXiv:170701836. 2017.

[17] Grewal M, Srivastava MM, Kumar P, Varadarajan S. RADNET: radiologist level accuracy using deep learning for HEMORRHAGE detection in CT scans. In: 2018 IEEE 15th international symposium on biomedical imaging (ISBI 2018). Washington, DC, USA: IEEE. 2018. p. 281–4. 10.1109/ISBI.2018.8363574.

[18] Demner-Fushman D, Kohli MD, Rosenman MB, Shooshan SE, Rodriguez L, Antani S (2016). Preparing a collection of radiology examinations for distribution and retrieval. JAMIA.

[19] Ryoo S, Kim HJ (2014). Activities of the Korean institute of tuberculosis. Osong Public Health Res Perspect.

[20] Jaeger S, Candemir S, Antani S, Wang YX, Lu PX, Thoma G (2014). Two public chest X-ray datasets for computer-aided screening of pulmonary diseases. Quant Imaging Med Surg.

[21] Shiraishi J, Katsuragawa S, Ikezoe J, Matsumoto T, Kobayashi T, Komatsu K (2000). Development of a digital image database for chest radiographs with and without a lung nodule: receiver operating characteristic analysis of radiologists’ detection of pulmonary nodules. AJR Am J Roentgenol.

[22] van Ginneken B, Stegmann MB, Loog M (2006). Segmentation of anatomical structures in chest radiographs using supervised methods: a comparative study on a public database. Med Image Anal.

[23] Wang XS, Peng YF, Lu L, Lu ZY, Bagheri M, Summers RM. ChestX-ray8: hospital-scale chest x-ray database and benchmarks on weakly-supervised classification and localization of common thorax diseases. In: 2017 IEEE conference on computer vision and pattern recognition (CVPR). Honolulu, HI, USA: IEEE. 2017. p. 3462–71. 10.1109/CVPR.2017.369.

[24] Sherrier RH, Johnson GA (1987). Regionally adaptive histogram equalization of the chest. IEEE Trans Med Imaging.

[25] Kwan B, Kwan HK. Improved lung nodule visualization on chest radiographs using digital filtering and contrast enhancement. World Acad Sci Technol. 2011; 110:590–3. http://waset.org/publications/6635.

[26] Xu X, Wang Y, Yang G, Hu Y. Image enhancement method based on fractional wavelet transform. In: 2016 IEEE international conference on signal and image processing (ICSIP). Beijing, China: IEEE. 2016. p. 194–7. 10.1109/SIPROCESS.2016.7888251.

[27] Savitha SK, Naveen NC. Algorithm for pre-processing chest-x-ray using multi-level enhancement operation. In: 2016 international conference on wireless communications, signal processing and networking (WiSPNET). Chennai, India: IEEE. 2016. p. 2182–6.

[28] Soleymanpour E, Pourreza HR, Ansaripour E, Yazdi MS (2011). Fully automatic lung segmentation and rib suppression methods to improve nodule detection in chest radiographs. J Med Signals Sens.

[29] Candemir S, Jaeger S, Palaniappan K, Musco JP, Singh RK, Xue Z (2014). Lung segmentation in chest radiographs using anatomical atlases with nonrigid registration. IEEE Trans Med Imaging.

[30] Candemir S, Jaeger S, Lin W, Xue Z, Antani S, Thoma G. Automatic heart localization and radiographic index computation in chest x-rays. In: Medical imaging 2016: computer-aided diagnosis. Vol. 9785. San Diego: SPIE; 2016. 10.1117/12.2217209.

[31] Zhanjun Y, Goshtasby A, Ackerman LV (1995). Automatic detection of rib borders in chest radiographs. IEEE Trans Med Imaging.

[32] Nakamori N, Doi K, Sabeti V, MacMahon H (1990). Image feature analysis and computer-aided diagnosis in digital radiography: automated analysis of sizes of heart and lung in chest images. Med Phys.

[33] Brown MS, Wilson LS, Doust BD, Gill RW, Sun C (1998). Knowledge-based method for segmentation and analysis of lung boundaries in chest X-ray images. Comput Med Imaging Graph.

[34] Cheng D, Goldberg M. An algorithm for segmenting chest radiographs. In: Proc SPIE. 1988. p. 261–8. 10.1117/12.968961.

[35] Armato SG, Giger ML, MacMahon H (1998). Automated lung segmentation in digitized posteroanterior chest radiographs. Acad Radiol.

[36] Li L, Zheng Y, Kallergi M, Clark RA (2001). Improved method for automatic identification of lung regions on chest radiographs. Acad Radiol.

[37] Iakovidis DK, Papamichalis G. Automatic segmentation of the lung fields in portable chest radiographs based on Bézier interpolation of salient control points. In: Proc IEEE Int Conf Img SysTech. Crete, Greece: IEEE. 2008. p. 82–7. 10.1109/IST.2008.4659946.

[38] Wan Ahmad WS, Zaki WM, Ahmad Fauzi MF (2015). Lung segmentation on standard and mobile chest radiographs using oriented Gaussian derivatives filter. Biomed Eng Online.

[39] Cootes TF, Taylor CJ, Cooper DH, Graham J (1995). Active shape models—their training and application. Comput Vis Image Underst.

[40] Cootes TF, Edwards GJ, Taylor CJ (2001). Active appearance models. IEEE Trans Pattern Anal.

[41] Li XC, Luo SH, Hu QM, Li JM, Wang DD, Chiong FB (2016). Automatic lung field segmentation in x-ray radiographs using statistical shape and appearance models. J Med Imaging Health Inf.

[42] van Ginneken B, Frangi AF, Staal JJ, Romeny BMT, Viergever MA (2002). Active shape model segmentation with optimal features. IEEE Trans Med Imaging.

[43] Iakovidis DK, Savelonas M. Active shape model aided by selective thresholding for lung field segmentation in chest radiographs. In: 2009 9th international conference on information technology and applications in biomedicine. Larnaca, Cyprus: IEEE. 2009. p. 1–4. 10.1109/itab.2009.5394326.

[44] Wu G, Zhang XD, Luo S, Hu QM (2015). Lung segmentation based on customized active shape model from digital radiography chest images. J Med Imaging Health Inf.

[45] Mcnittgray MF. Pattern classification approach to segmentation of chest radiographs. In: Medical imaging 1993; Newport Beach, CA, United States. SPIE. 1993. p. 160–70. 10.1117/12.154500.

[46] Vittitoe NF, Vargas-Voracek R, Floyd CE (1998). Identification of lung regions in chest radiographs using Markov random field modeling. Med Phys.

[47] Shi Z, Zhou P, He L, Nakamura T, Yao Q, Itoh H. Lung segmentation in chest radiographs by means of Gaussian Kernel-based FCM with spatial constraints. In: 2009 sixth international conference on fuzzy systems and knowledge discovery; Tianjin, China. IEEE. 2009. p. 428–32. 10.1109/FSKD.2009.811.

[48] Shelhamer E, Long J, Darrell T (2017). Fully convolutional networks for semantic segmentation. IEEE Trans Pattern Anal Mach Intell.

[49] Badrinarayanan V, Handa A, Cipolla R. SegNet: a deep convolutional encoder-decoder architecture for robust semantic pixel-wise labelling. arXiv preprint arXiv:150507293. 2015. 10.1109/TPAMI.2016.2644615 28060704

[50] Ronneberger O, Fischer P, Brox T. U-Net: convolutional networks for biomedical image segmentation. In: Medical image computing and computer-assisted intervention: MICCAI international conference on medical image computing and computer-assisted intervention. 2015. p. 234–41.

[51] Novikov AA, Lenis D, Major D, Hladůvka J, Wimmer M, Bühler K (2017). Fully convolutional architectures for multi-class segmentation in chest radiographs. IEEE Trans Med Imaging.

[52] Dai W, Doyle J, Liang X, Zhang H, Dong N, Li Y, et al. SCAN: Structure Correcting Adversarial Network for organ segmentation in chest x-rays. arXiv preprint arXiv:170308770. 2017.

[53] Suzuki K, Abe H, MacMahon H, Doi K (2006). Image-processing technique for suppressing ribs in chest radiographs by means of massive training artificial neural network (MTANN). IEEE Trans Med Imaging.

[54] Loog M, Ginneken BV. Bony structure suppression in chest radiographs. In: computer vision approaches to medical image analysis. Vol. 4241. Graz, Austria: Springer. 2006. p. 166–77. 10.1007/11889762_15.

[55] Freedman MT, Lo SCB, Seibel JC, Bromley CM (2011). Lung nodules: improved detection with software that suppresses the rib and clavicle on chest radiographs. Radiology.

[56] Oda S, Awai K, Suzuki K, Yanaga Y, Funama Y, MacMahon H (2009). Performance of radiologists in detection of small pulmonary nodules on chest radiographs: effect of rib suppression with a massive-training artificial neural network. AJR Am J Roentgenol.

[57] Li F, Engelmann R, Pesce LL, Doi K, Metz CE, MacMahon H (2011). Small lung cancers: improved detection by use of bone suppression imaging-comparison with dual-energy subtraction chest radiography. Radiology.

[58] Li F, Engelmann R, Pesce L, Armato SG, Macmahon H (2012). Improved detection of focal pneumonia by chest radiography with bone suppression imaging. Eur Radiol.

[59] Vock P, Szucs-Farkas Z (2009). Dual energy subtraction: principles and clinical applications. Eur J Radiol.

[60] Nguyen HX, Dang TT. Ribs suppression in chest x-ray images by using ICA method. In: Van Toi V, Tran PHL, editors. Ifmbe Proc; Cham: Springer. 2015. p. 194–7. 10.1007/978-3-319-11776-8_47.

[61] Yang W, Chen Y, Liu Y, Zhong L, Qin G, Lu Z (2017). Cascade of multi-scale convolutional neural networks for bone suppression of chest radiographs in gradient domain. Med Image Anal.

[62] Gordienko Y, Peng G, Jiang H, Wei Z, Kochura Y, Alienin O, et al. Deep learning with lung segmentation and bone shadow exclusion techniques for chest x-ray analysis of lung cancer. arXiv preprint arXiv:171207632. 2017.

[63] Jaeger S, Karargyris A, Candemir S, Siegelman J, Folio L, Antani S (2013). Automatic screening for tuberculosis in chest radiographs: a survey. Quant Imaging Med Surg.

[64] Stewart BW, Wild CP. World cancer report 2014. World. http://www.who.int/cancer/publications/WRC_2014/en/. Accessed 10 June 2018.

[65] Chen S, Suzuki K, MacMahon H (2011). Development and evaluation of a computer-aided diagnostic scheme for lung nodule detection in chest radiographs by means of two-stage nodule enhancement with support vector classification. Med Phys.

[66] Kobayashi T, Xu XW, MacMahon H, Metz CE, Doi K (1996). Effect of a computer-aided diagnosis scheme on radiologists’ performance in detection of lung nodules on radiographs. Radiology.

[67] Schilham AM, van Ginneken B, Loog M (2006). A computer-aided diagnosis system for detection of lung nodules in chest radiographs with an evaluation on a public database. Med Image Anal.

[68] Hassen DB, Taleb H (2013). Automatic detection of lesions in lung regions that are segmented using spatial relations. Clin Imaging.

[69] Al-Absi HRH, Samir BB, Sulaiman S (2014). A computer aided diagnosis system for lung cancer based on statistical and machine learning techniques. J Comput.

[70] Wei J, Hagihara Y, Shimizu A, Kobatake H. Optimal image feature set for detecting lung nodules on chest X-ray images. In: CARS 2002 computer assisted radiology and surgery. Berlin: Springer; 2002. p. 706–11. 10.1007/978-3-642-56168-9_118.

[71] Shiraishi J, Li Q, Suzuki K, Engelmann R, Doi K (2006). Computer-aided diagnostic scheme for the detection of lung nodules on chest radiographs: localized search method based on anatomical classification. Med Phys.

[72] Hardie RC, Rogers SK, Wilson T, Rogers A (2008). Performance analysis of a new computer aided detection system for identifying lung nodules on chest radiographs. Med Image Anal.

[73] Oğul BB, Koşucu P, Özçam A, Kanik SD. Lung nodule detection in x-ray images: a new feature set. In: European conference of the international federation for medical and biological engineering; Cham: Springer International Publishing; 2015. p. 150–5.

[74] Pan SJ, Yang Q (2010). A survey on transfer learning. IEEE Trans Knowl Data Eng.

[75] Bar Y, Diamant I, Wolf L, Greenspan H. Deep learning with non-medical training used for chest pathology identification. In: SPIE medical imaging; Orlando, Florida, United States. SPIE; 2015. p. 7. 10.1117/12.2083124.

[76] Bush I. Lung nodule detection and classification 2016. http://cs231n.stanford.edu/reports/2016/pdfs/313_Report.pdf. Accessed 20 Apr 2018.

[77] Wang CM, Elazab A, Wu JH, Hu QM (2017). Lung nodule classification using deep feature fusion in chest radiography. Comput Med Imaging Graph.

[78] WHO. Global tuberculosis report 2017. 2017. http://www.who.int/tb/publications/global_report/en/. Accessed 11 June 2018.

[79] Roy M, Ellis S (1021). Radiological diagnosis and follow-up of pulmonary tuberculosis. Postgrad Med J.

[80] Rohmah RN, Susanto A, Soesanti I. Lung tuberculosis identification based on statistical feature of thoracic X-ray. In: 2013 international conference on QiR; Yogyakarta, Indonesia. IEEE. 2013. p. 19–26. 10.1109/QiR.2013.6632528.

[81] Tan JH, Acharya UR, Tan C, Abraham KT, Lim CM (2012). Computer-assisted diagnosis of tuberculosis: a first order statistical approach to chest radiograph. J Med Syst.

[82] Noor NM, Rijal OM, Yunus A, Mahayiddin AA, Peng GC, Abu-Bakar SAR. A statistical interpretation of the chest radiograph for the detection of pulmonary tuberculosis. In: 2010 IEEE EMBS conference on biomedical engineering and sciences (IECBES); Kuala Lumpur, Malaysia. IEEE. 2010. p. 47–51. 10.1109/iecbes.2010.5742197.

[83] Leibstein JM, Nel AL. Detecting tuberculosis in chest radiographs using image processing techniques. University of Johannesburg. 2006. http://www.satnac.org.za/proceedings/2011/papers/Posters/235.pdf

[84] Maduskar P, Hogeweg L, Philipsen R, Schalekamp S, van Ginneken B. Improved texture analysis for automatic detection of tuberculosis (TB) on chest radiographs with bone suppression images. In: Medical imaging 2013: Computer-aided diagnosis. Vol. 8670. Lake Buena Vista (Orlando Area), Florida, United States: SPIE. 2013. Vol. 1. p. 148–9. 10.1117/12.2008083.

[85] Hogeweg L, Mol C, de Jong PA, Dawson R, Ayles H, van Ginneken B (2010). Fusion of local and global detection systems to detect tuberculosis in chest radiographs. Med Image Comput Comput Assist Interv.

[86] Hogeweg L, Sanchez CI, Maduskar P, Philipsen R, Story A, Dawson R (2015). Automatic detection of tuberculosis in chest radiographs using a combination of textural, focal, and shape abnormality analysis. IEEE Trans Med Imaging.

[87] Song YL, Yang Y. Localization algorithm and implementation for focal of pulmonary tuberculosis chest image. In: 2010 international conference on machine vision and human–machine interface. Kaifeng, China: IEEE. 2010. p. 361–4. 10.1109/mvhi.2010.180.

[88] Shen R, Cheng I, Basu A (2010). A hybrid knowledge-guided detection technique for screening of infectious pulmonary tuberculosis from chest radiographs. IEEE Trans Biomed Eng.

[89] Xu T, Cheng I, Long R, Mandal M (2013). Novel coarse-to-fine dual scale technique for tuberculosis cavity detection in chest radiographs. EURASIP J Image Video Process.

[90] Hwang S, Kim HE, Jeong J. A novel approach for tuberculosis screening based on deep convolutional neural networks. In: Medical imaging 2016: computer-aided diagnosis. Vol. 9785. San Diego: SPIE; 2016. 10.1117/12.2216198.

[91] Lakhani P, Sundaram B (2017). Deep learning at chest radiography: automated classification of pulmonary tuberculosis by using convolutional neural networks. Radiology.

[92] Arzhaeva Y, Tax D, van Ginneken B. Improving computer-aided diagnosis of interstitial disease in chest radiographs by combining one-class and two-class classifiers. In: Medical imaging 2006: image processing. Vol. 6144. San Diego: SPIE; 2006. 10.1117/12.652208.

[93] Miller WT (2002). Radiographic evaluation of diffuse interstitial lung disease: review of a dying art. Semin Ultrasound CT MRI.

[94] Plankis T, Juozapavicius A, Stasiene E, Usonis V (2017). Computer-aided detection of interstitial lung diseases: a texture approach. Nonlin Anal Model.

[95] Antoniou KM, Margaritopoulos GA, Tomassetti S, Bonella F, Costabel U, Poletti V (2014). Interstitial lung disease. Eur Respir Rev.

[96] Katsuragawa S, Doi K, MacMahon H (1989). Image feature analysis and computer-aided diagnosis in digital radiography: classification of normal and abnormal lungs with interstitial disease in chest images. Med Phys.

[97] Kido S, Ikezoe J, Naito H, Tamura S, Machi S (1995). Fractal analysis of interstitial lung abnormalities in chest radiography. Radiographics.

[98] Ishida T, Katsuragawa S, Kobayashi T, MacMahon H, Doi K (1997). Computerized analysis of interstitial disease in chest radiographs: improvement of geometric-pattern feature analysis. Med Phys.

[99] Loog M, van Ginneken B, Nielsen M. Detection of interstitial lung disease in PA chest radiographs. In: Medical imaging 2004: physics of medical imaging. Vol. 5368. San Diego: SPIE; 2004. 10.1117/12.535307.

[100] Abe H, Macmahon H, Shiraishi J, Li Q, Engelmann R, Doi K (2004). Computer-aided diagnosis in chest radiology. Semin Ultrasound CT MRI.

[101] Islam MT, Aowal MA, Minhaz AT, Ashraf K. Abnormality detection and localization in chest x-rays using deep convolutional neural networks. arXiv preprint arXiv:170509850. 2017.

[102] Parveen NR, Sathik MM (2011). Detection of pneumonia in chest X-ray images. J X-ray Sci Technol.

[103] Kumar A, Yen-Yu W, Kai-Che L, Tsai IC, Ching-Chun H, Nguyen H. Distinguishing normal and pulmonary edema chest x-ray using Gabor filter and SVM. In: 2014 IEEE international symposium on bioelectronics and bioinformatics (IEEE ISBB 2014). Chung Li, Taiwan: IEEE. 2014. p. 1–4. 10.1109/isbb.2014.6820918.

[104] Avni U, Greenspan H, Konen E, Sharon M, Goldberger J (2011). X-ray categorization and retrieval on the organ and pathology level, using patch-based visual words. IEEE Trans Med Imaging.

[105] Noor NM, Rijal OM, Yunus A, Mahayiddin AA, Gan CP, Ong EL, Lai KW, Hum YC, Mohamad Salim MI, Ong S-B, Utama NP, Myint YM, Mohd Noor N, Supriyanto E (2014). Texture-based statistical detection and discrimination of some respiratory diseases using chest radiograph. Advances in medical diagnostic technology.

[106] Cicero M, Bilbily A, Colak E, Dowdell T, Gray B, Perampaladas K (2017). Training and validating a deep convolutional neural network for computer-aided detection and classification of abnormalities on frontal chest radiographs. Invest Radiol.

[107] Yao L, Poblenz E, Dagunts D, Covington B, Bernard D, Lyman K. Learning to diagnose from scratch by exploiting dependencies among labels. arXiv preprint arXiv:171010501. 2017.

[108] Huang G, Liu Z, Maaten Lvd, Weinberger KQ. Densely Connected Convolutional Networks. In: 2017 IEEE conference on computer vision and pattern recognition (CVPR). Honolulu: IEEE; 2017. p. 4700–8. 10.1109/cvpr.2017.243.

[109] Ioffe S, Szegedy C. Batch normalization: accelerating deep network training by reducing internal covariate shift. In: International conference on machine learning. 2015. p. 448–56.

[110] Kumar P, Grewal M, Srivastava MM. Boosted cascaded convnets for multilabel classification of thoracic diseases in chest radiographs. arXiv preprint arXiv:171108760. 2017.

[111] Guan Q, Huang Y, Zhong Z, Zheng Z, Zheng L, Yang Y. Diagnose like a radiologist: attention guided convolutional neural network for thorax disease classification. arXiv preprint arXiv:180109927. 2018.

[112] What’s the difference between artificial intelligence, machine learning, and deep learning? https://blogs.nvidia.com/blog/2016/07/29/whats-difference-artificial-intelligence-machine-learning-deep-learning-ai/. Accessed 11 June 2018.

[113] Chen Y, Li J, Xiao H, Jin X, Yan S, Feng J. Dual path networks. In: Neural information processing systems (NIPS). 2017.

[114] Wang F, Jiang M, Qian C, Yang S, Li C, Zhang H, et al. Residual attention network for image classification. In: 2017 IEEE conference on computer vision and pattern recognition (CVPR). Honolulu, HI, USA: IEEE. 2017. p. 6450–8.

[115] Huang R, Zhang S, Li T, He R. Beyond Face Rotation: Global and Local Perception GAN for Photorealistic and Identity Preserving Frontal View Synthesis. In: 2017 IEEE international conference on computer vision (ICCV). Venice, Italy; 2018. p. 2458–67. https://doi.ieeecomputersociety.org/10.1109/iccv.2017.267.

[116] Silver D, Schrittwieser J, Simonyan K, Antonoglou I, Huang A, Guez A (2017). Mastering the game of Go without human knowledge. Nature.

